# No evidence for human or rodent deltavirus replication in yeast

**DOI:** 10.17912/micropub.biology.001743

**Published:** 2025-10-03

**Authors:** Louison Bois Naegelin, Valérie Courgnaud, Dominique Helmlinger, Karim Majzoub

**Affiliations:** 1 Institut de Génétique Moléculaire de Montpellier, Montpellier, Occitanie, France; 2 Centre de Recherche en Biologie cellulaire de Montpellier, Montpellier, Occitanie, France; 3 Centre National de la Recherche Scientifique, Paris, Île-de-France, France; 4 Université de Montpellier, Montpellier, Occitanie, France

## Abstract

Deltaviruses were recently discovered across a broad range of metazoan species. Their genome encodes a single protein and thus requires host factors for replication, which are likely evolutionarily conserved given their broad host range. Here we describe a genetic tool to unambiguously determine whether an RNA virus replicates in model yeast species. Our system involves transcriptional induction of viral RNA from an integrated cDNA template followed by its genomic excision. Testing Hepatitis D and Rodent delta viruses revealed that neither viral RNAs can replicate in yeasts, suggesting that Ascomycetes lack factors essential for their RNA-dependent amplification

**Figure 1. Testing human and rodent deltaviruses expression and replication in Saccharomyces cerevisiae and Schizosaccharomyces pombe:**
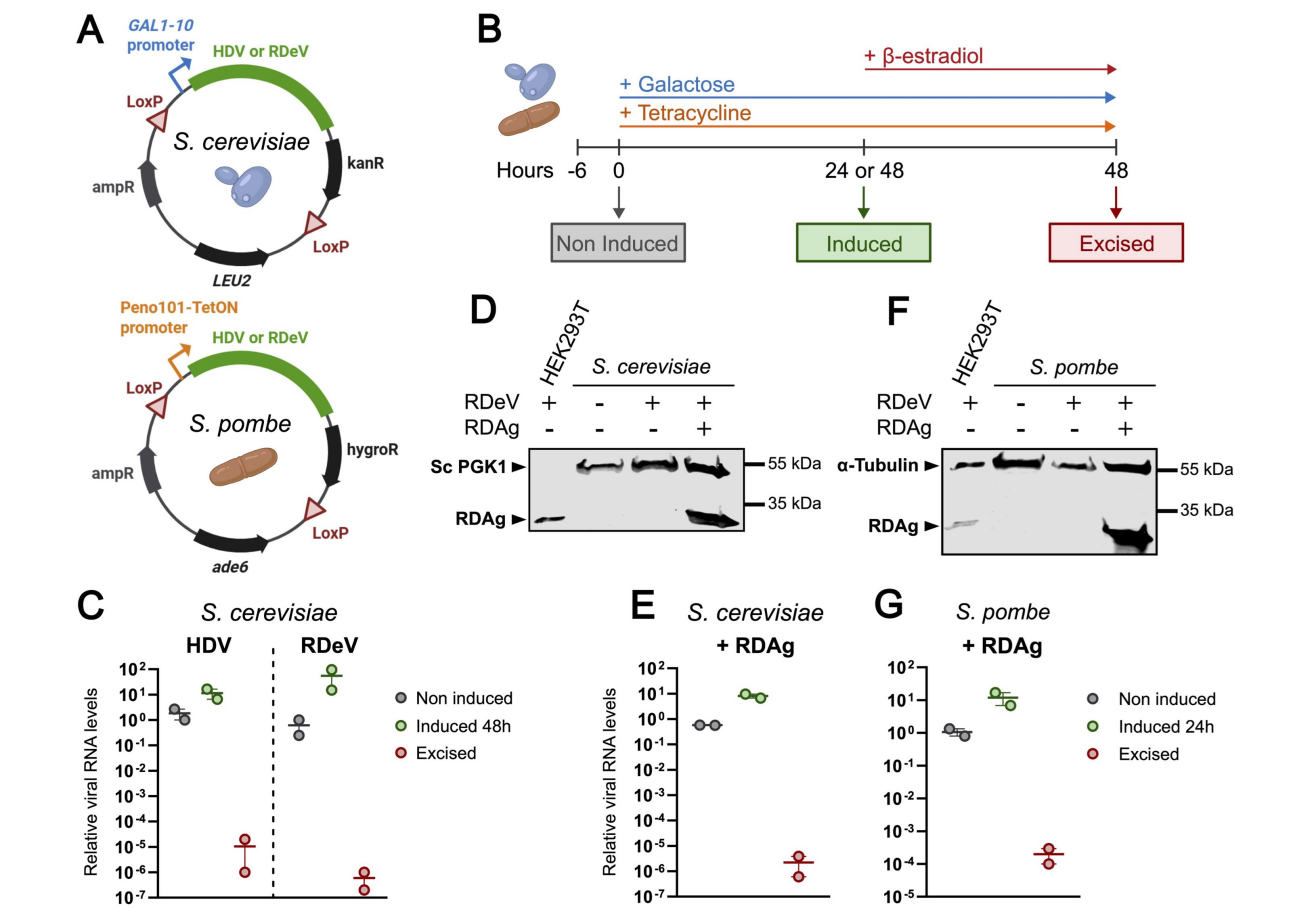
**A)** Plasmid maps of DNA used to transform *S. cerevisiae* (top) and *S. pombe* (bottom) containing viral cDNA. **B)** Schematics of the experimental design. Yeast cultures were collected in exponential phase before induction, 24 or 48 hours post-induction, and 48h post-β-estradiol treatment to excise viral cDNA. **C)** Quantitative RT-PCR analyses using primers targeting HDV (left) or RDeV (right) RNA in *S. cerevisiae* . **D)** Western blot analysis of RDAg and *S. cerevisiae* Pgk1 in strains with or without integrated RDeV cDNA and an episomal copy of RDAg. **E)** Quantitative RT-PCR analysis using primers targeting non-coding region of the RDeV RNA in *S. cerevisiae* RDeV strains expressing RDAg. **F)** Western blot analysis of RDAg and α-Tubulin in *S. pombe* strains with or without integrated RDeV cDNA and an episomal copy of RDAg. **D,F** ) HEK293T transfected with RDeV cDNA are shown as a positive control for RDAg detection. **G)** Quantitative RT-PCR analysis using primers targeting the non-coding region of the RDeV RNA in *S. pombe* RDeV strains expressing RDAg.

## Description

Recent years have witnessed the discovery of Hepatitis D virus- *like* sequences in a wide range of animal species, including rats, bats, snakes, frogs, birds, and termites (Bergner et al., 2021; W.-S. Chang et al., 2019; Hetzel et al., 2019; Iwamoto et al., 2021; Paraskevopoulou et al., 2020; Wille et al., 2018). This led to the establishment of the viral family *Kolmioviridae* , also known as deltaviruses (Hepojoki et al., 2021). Deltaviruses are the smallest satellite viruses known to infect animals. Their genome is a single-stranded negative-sense circular RNA of approximately 1.7 kb (Pérez-Vargas et al., 2021). The most studied deltavirus, Hepatitis D Virus (HDV), infects humans and causes the most severe form of viral hepatitis (Khalfi et al., 2023). Because these viruses infect a broad range of animal species, their replication depends on highly conserved mechanisms (Lehmann et al., 2007). Indeed, several studies have shown that both HDV and rodent deltavirus (RDeV) are able to replicate in heterologous animal cells (Gnouamozi et al., 2024; Khalfi et al., 2024; Paraskevopoulou et al., 2020). Whether these viruses can replicate in other eukaryotic kingdoms of life has never been reported. To address this question, we tested the ability of HDV and RDeV to replicate in the model organism *Saccharomyces cerevisiae* . The budding yeast is indeed one of the most well-understood eukaryotic organisms and has already proven useful in virology. Notably, the yeast two-hybrid system was used to characterize HIV-1 protein interactions with host factors (Andréola & Litvak, 2012).

Two copies of the cDNA from either the HDV or RDeV genome were cloned in head-to-tail orientation, as described before (Paraskevopoulou et al., 2020), into plasmids enabling stable genomic integration at the *LEU2* gene locus. The viral cDNAs were placed under the control of the *GAL1-10* promoter and before an antibiotic selection marker, generating an inducible and selectable expression module, which we flanked with LoxP sites for Cre-mediated excision ( Fig. 1A, B). The obtained plasmids were transformed into a strain stably expressing a β-estradiol-inducible Cre-ER recombinase (Verzijlbergen et al., 2010), allowing controllable excision of the viral sequence through homologous recombination ( Fig. 1B ).

We first analyzed HDV and RDeV RNA levels upon induction and excision of the viral cDNA by RT-qPCR ( Fig. 1C ). Viral RNA levels showed a 10-fold increase upon galactose induction ( Fig. 1C ). Following template excision and clone propagation, viral RNA decreased substantially, to reach the detection limit of qPCR, indicating that viral RNA is unable to replicate and persist in the absence of its cDNA template. The viral RNA detected before induction ( Fig. 1C ) is most likely due to low *GAL1-10* promoter activity in raffinose media (non-induced in Fig. 1B ).

The delta-antigen (DAg) is essential for replication and forms viral ribonucleoproteins in association with the RNA genome. DAg proteins are imported into the nuclei of infected cells and recruit host RNA polymerase II to initiate viral replication (J. Chang et al., 2008a; Lehmann et al., 2007). We thus hypothesized that HDV and RDeV might be unable to replicate because the deltavirus strains we used lack DAg, possibly because induced viral RNAs are not processed into a DAg mRNA that is matured for export and translation. To test if exogenous addition of the viral DAg protein triggers viral replication, we transformed a non-integrative plasmid encoding the rodent DAg (RDAg) into the RDeV strains. Western blot analyses showed that RDeV strains lacked RDAg expression, even when RDeV genome expression was induced ( Fig. 1D ), while RDAg was detected in RDeV strains transformed with the RDAg encoding plasmid ( Fig. 1D ). We then monitored RDeV RNA genome levels by RT-qPCR and observed an increase of viral RNA levels upon induction and a decrease after viral cDNA excision, mirroring what we found in RDeV strains lacking the RDAg ( Fig. 1E ). We conclude that RDeV is unable to replicate in *S. cerevisiae* , even in the presence of exogenous RDAg protein.

Our results suggest that *S. cerevisiae* lacks one or more host factors essential for HDV and RDeV replication. *Schizosaccharomyces pombe* is another prime model yeast species that diverged from other Ascomycetes at the root of this clade, about 530 million years ago (Shen et al., 2020). Thus, comparing these two model species can bring complementary information on fundamental biological processes.

To determine whether *S. pombe* is able to sustain deltavirus replication, the RDeV cDNA was cloned as described before, in a plasmid for inducible expression and controllable excision in *S. pombe* . For this, we used a recently available vector containing a tetracycline-inducible promoter (Lyu et al., 2024), a constitutively expressed Tet repressor cDNA, and a homologous region for stable integration upstream of the *ade6* gene locus. The entire viral expression module was flanked by two LoxP sites for β-estradiol-inducible Cre-mediated excision ( Fig. 1A ). An *S. pombe* strain stably expressing the Cre-ER recombinase was transformed and analyzed as schematized in Fig. 1B, analogous to *S. cerevisiae* . Western blot analysis showed that the RDAg was not expressed upon viral genome induction with tetracycline ( Fig. 1F ). We therefore transformed this RDeV strain with a non-integrative plasmid encoding RDAg to directly assess the replication in the presence of the delta-antigen, which expression was confirmed by Western blot ( Fig. 1F ). In this strain, while the levels of RDeV viral RNA increased by 10-fold upon tetracycline induction, they decreased to barely detectable levels upon RDeV cDNA excision ( Fig. 1G ). These results indicate that RDeV cannot replicate in *S. pombe* , even when the RDAg is present, as in *S. cerevisiae* ( Fig. 1E ).

In conclusion, our study suggests that despite the broad range of species infected by deltaviruses, their replication appears restricted to animals, suggesting that deltavirus replication requires metazoan-specific co-factors, for example for recruiting or activating the RNA polymerase II machinery on an RNA template (J. Chang et al., 2008b; Lehmann et al., 2007). Although *S. cerevisiae* RNA polymerase II has RNA-dependent RNA polymerase (RdRP) activity on HDV RNA *in vitro* (Lehmann et al., 2007), our results demonstrate that other factors might be required for viral RNA amplification *in vivo* . In addition, other essential pro-viral host factors involved in the deltavirus lifecycle may be lacking in Ascomycetes, such as an RNA ligase (Reid & Lazinski, 2000). Another possibility explaining the non-permissiveness of yeasts to deltavirus replication is the expression of antiviral factors that might repress viral replication. For instance, the SKI complex is important for Killer virus attenuation in *S. cerevisiae* and is present in *S. pombe* . Similarly, a conserved RNAi machinery exists in *S. pombe* and might inhibit deltavirus replication in this species, as RNAi has a potent antiviral function in plants and possibly fungi (S.-S. Chang et al., 2012; Ding & Voinnet, 2007). Importantly, our work provides a novel methodological framework for the rigorous assessment of circular RNA virus replication in yeast, through sequential transcriptional induction and recombination-mediated excision of a viral cDNA template. This issue is important because previous studies may have mistakenly interpreted residual viral RNA levels as evidence of autonomous replication, which we show completely disappeared upon loss of the cDNA template for deltaviruses (Delan-Forino et al., 2011; Janda, 1993).

## Methods

Cloning:

Human and rodent deltavirus genomes and antigen-coding sequences were amplified from previously described pcDNA3.1 plasmids (Khalfi et al., 2024) and cloned in expression plasmids for *S. cerevisiae* and *S. pombe* by Gibson Assembly (New England Biolabs).

*S. cerevisiae* and *S. pombe* :

All *S. cerevisiae* and *S. pombe* strains are listed in Table 1. *S. cerevisiae* was grown at 30°C in rich YEPD medium for parental, HDV, and RDeV strains, and in synthetic minimal YNB without histidine for RDAg strains. *S. pombe* was grown at 32°C in rich YES medium for parental and RDeV strains, and in synthetic minimal EMM without uracil for RDAg strains. Yeasts were transformed using a LiAc-based procedure for chemical competence (Gietz et al., 1992). HDV and RDeV cDNA integration was selected on YNB without leucine or YEPD with G418 for *S. cerevisiae* and on EMM without adenine or YES with hygromycin for *S. pombe* . *S. cerevisiae* harboring RDAg coding plasmids were selected and kept on YNB without histidine, while *S. pombe* harboring RDAg coding plasmids were selected and kept on EMM without uracil.

Viral genome induction:

Viral genome expression was induced with 2% galactose for *S. cerevisiae* and with 2.5 µg.mL ^-1^ tetracycline for *S. pombe* (Lyu et al., 2024). Cre-ER recombinase was activated with 1µM β-estradiol. Excision was validated on a rich medium plate containing G418 or hygromycin for *S. cerevisiae* and *S. pombe* , respectively. For each sample, 50 mL of exponentially growing cells were collected, pelleted, and stored at -80°C before RNA or protein extraction.

RT-qPCR:

RNA extraction was performed using hot acidic phenol as described previously (Toullec et al., 2021). and DNase I digestion was used to remove contaminating DNA. 1 µg of RNA was reverse transcribed with SuperScript III (Invitrogen). Fluorescence-based quantitative PCR was performed using SYBR Green on the CFX Opus 384 Real-Time PCR System (Bio-Rad), and relative quantities were calculated from standard curves.

Western blot:

Western blot was then performed as described before (Khalfi et al., 2024), using the same secondary antibodies. *S. cerevisiae* was incubated in 0.1M NaOH for 5 minutes, pelleted, and resuspended in protein loading dye. Samples were then denatured for 3 minutes at 95°C, pelleted, and the supernatant was used for Western blot. An anti-Pgk1 antibody was used to control for loading (ThermoFisher #459250). RDAg was detected using polyclonal antibodies extracted from patient sera (Khalfi et al., 2024).

For *S. pombe* , protein extraction was performed using lysis buffer (40 mM HEPES-NaOH pH 7.4, 350 mM NaCl, 0.1% NP40, and 10% glycerol) supplemented with protease inhibitors (cOmplete EDTA-free cocktails tablets, 1 mM PMSF, 1 mg.ml ^-1^ bestatin, and 1 mg.ml ^-1^ pepstatin A). An anti-α-Tubulin antibody was used to control for loading. RDAg was detected using rabbit serum/antiserum immunized with recombinant SDAg (Hetzel et al., 2019).

## Reagents

**Table:** 

**Strain**	**Genotype**	**Source**
DHP1801 *S. cerevisiae* parental	*ade2-1 trp1-1 can1-100 leu2-3,112 his3-11,15 ura3-1 psi+ pRS306-CreEBD78-URA3 MATα*	Lab stock
DHP1802 *S. cerevisiae* HDV	*ade2-1 trp1-1 can1-100 his3-11,15 ura3-1 psi+ pRS306-CreEBD78-URA3 DHB403 MATα*	This study
DHP1803 *S. cerevisiae* RDeV	*ade2-1 trp1-1 can1-100 his3-11,15 ura3-1 psi+ pRS306-CreEBD78-URA3 DHB402 MATα*	This study
DHP1804 *S. cerevisiae* RDeV + RDAg	*ade2-1 trp1-1 can1-100 his3-11,15 ura3-1 psi+ pRS306-CreEBD78 DHB402 OKM80 MATα*	This study
DHP1805 *S. pombe* parental	*ade6-M210 leu1-32 ars1::pRad15-CreEBD78-leu2 h+*	Lab stock
DHP1806 *S. pombe* RDeV	*ade6-M210 leu1-32 ars1::pRad15-CreEBD78-leu2 DHB404 h+*	This study
DHP1807 *S. pombe* RDeV + RDAg	*ade6-M210 leu1-32 ura4-D18 ars1::pRad15-CreEBD78-leu2 DHB404 DHB405 h+*	This study

**Table:** 

**qPCR oligonucleotides**	**Sequence**
OKM690 HDV forward	5’-CTCGGTCAACCTCCTGAGTT-3’
OKM691 HDV reverse	5’-AAGGCCCTCGAGAACAAGAA-3’
OKM678 RDeV forward	5’-CCATATTTCCAACAGCCGGG-3’
OKM679 RDeV reverse	5’-AAGGAGGGAGAGGGGAAAAC-3’

**Table:** 

**Plasmid name**	**Main features**
DHB331	pD371-loxP-kanMX6-GAL1-10-loxP-ScADH1term
DHB402	pD371-loxP-kanMX6-GAL1-10-RDeV2X-loxP-ScADH1term
DHB403	pD371-loxP-kanMX6-GAL1-10-HDV2X-loxP-ScADH1term
DHB332	pDB5320-ade6-NotI-CMV-tetR-loxP-enotetSW2-ScADH1term-hphMX6-loxP
DHB404	pDB5320-ade6-NotI-CMV-tetR-loxP-enotetSW2-RDAg-ScADH1term-hphMX6-loxP
DHB401	pSP403-HIS3-CEN/ARS-TDH3prom-CYC1term
OKM80	pSP403-HIS3-CEN/ARS-TDH3prom-RDAg-CYC1term
DHB389	pREP1NT-ura4-ars1-nmt1prom
DHB405	pREP1NT-ura4-ars1-nmt1prom-RDAg
